# Weight Loss, but Not at Any Cost: Risks and Challenges in Patients with Osteoarthritis

**DOI:** 10.31138/mjr.121224.wlc

**Published:** 2025-03-31

**Authors:** Tsvetoslav Georgiev, Plamena Kabakchieva

**Affiliations:** 1 Clinic of Rheumatology, University Hospital “St. Marina”, Varna, Bulgaria; 2 First Department of Internal Medicine, Faculty of Medicine, Medical University Varna, Varna, Bulgaria; 3 Clinic of Internal Diseases, Naval Hospital Varna, Military Medical Academy, Bulgaria

**Keywords:** osteoarthritis, weight loss, weight management, side effects, mental health

## Abstract

Osteoarthritis (OA) is a prevalent joint disorder characterised by the deterioration of the entire joint. Among its primary risk factors, obesity significantly contributes to OA onset and progression. Weight reduction in individuals with OA can alleviate pain, enhance joint function, and potentially delay or prevent the need for surgical interventions. However, despite these benefits, the potential risks and detriments associated with weight loss in OA patients warrant careful evaluation. This review synthesises available data on the multifaceted effects of weight loss interventions in OA patients, including risks of weight regain, malnutrition, sarcopenia, joint instability, bone density reduction, and psychoemotional stress due to fluctuating weight. A comprehensive search was conducted across major databases, identifying studies that assessed the physical, mental, and quality of life impacts of weight loss in knee and hip OA populations. Rapid weight loss may destabilise joints, lead to muscle and bone loss, and increase the risk of malnutrition and osteoporosis. Additionally, psychological distress from weight loss failures or fluctuations can adversely affect mental health and quality of life, underscoring the need for balanced weight management strategies. Long-term weight loss maintenance remains a challenge, with high rates of weight regain observed in OA patients. Emerging anti-obesity drugs hold potential for more sustained outcomes, albeit with uncertainties remaining. By adopting a holistic approach that addresses both physical and mental aspects, healthcare providers can improve outcomes and quality of life for OA patients, tailoring strategies to reduce the potential harms associated with aggressive or unsupervised weight reduction efforts.

## INTRODUCTION

Osteoarthritis (OA), the most prevalent joint disorder among the elderly, is a chronic condition that results in articular cartilage injury, accompanied by low-grade inflammation and adjacent bone remodelling.^1^ The knee and hip joints are commonly affected, causing significant pain, stiffness, and decreased mobility. One of the main risk factors for the onset and advancement of osteoarthritis in the knee and hip is obesity.^2^ As a result, weight loss has been widely advocated as a strategy to manage the symptoms of this debilitating condition.^3–5^ Indisputably, weight loss has been shown to have significant positive effects on knee and hip osteoarthritis. By reducing the load on the affected joints, weight loss can decrease pain levels, improve joint function,^6– 8^ and slow the progression of the disease.^9^ Meaningful weight reduction may decrease the risk of total knee replacement, while maintaining an appropriate weight could delay the need for hip arthroplasty.^10^ On another note, inflammation plays a key role in the development and progression of OA, and obesity has been shown to contribute to the chronic low-grade inflammation associated with the condition. Adipose tissue dysfunction and overexpression of various proinflammatory cytokines, known as adipocytokines, affect OA-associated pain and stiffness through diverse mechanisms promoting nociceptive pain, peripheral and central sensitisation.^11^

While weight loss can significantly benefit individuals with knee and hip osteoarthritis, optimising its effectiveness across individual, group, and population levels requires careful consideration of potential risks. Approaching weight reduction in osteoarthritis patients should involve close healthcare supervision to prevent unintended consequences, such as muscle and bone loss, joint instability, malnutrition, and psychoemotional stress linked to frequent weight fluctuations. Additionally, strategies to enhance long-term effectiveness are essential to minimise the likelihood of weight regain following initial weight loss.

Hence, it is of paramount importance to critically evaluate and synthesise the available evidence on the potential negative consequences or adverse events that may occur as a result of weight loss interventions or advice, including those related to physical health, mental health, and quality of life.

## SEARCH STRATEGY

To evaluate the potential negative consequences, risks or adverse outcomes of weight loss interventions, a comprehensive search strategy was employed in line with recommendations of Gasparyan et al. for writing a narrative biomedical review.^12^ This included searching Medline/PubMed and Scopus databases for relevant studies that assessed the impact of weight loss interventions on physical health, mental health, and quality of life outcomes in patients with knee or hip osteoarthritis but also in wider population groups. The search yielded a significant number of studies that provided valuable insights into the potential risks and benefits associated with these interventions. These findings will be synthesised to provide a comprehensive understanding of the overall impact of weight-loss interventions on individuals’ well-being. Here, we choose the form of narrative review because it allows for a flexible synthesis of diverse evidence, integrating findings from various study designs to explore the multifaceted risks, challenges, and clinical implications of weight-loss interventions.

## LONG-TERM DATA ON WEIGHT SUSTAINABILITY AND WEIGHT REGAIN AS A CHALLENGE

Weight regain after a successful weight loss intervention is a common side effect.^13^ Studies have shown that a considerable portion of individuals who lose weight are unable to maintain their weight loss long-term and often regain much of the weight they lost.^14^ Furthermore, a recent study highlights the concept of “obesogenic memory”, rooted primarily in stable epigenetic modifications within adipocytes and potentially other cell types. These changes appear to predispose cells to pathological responses in obesogenic environments, offering a possible explanation for the challenging “yo-yo” effect frequently observed with dieting.^15^ Studies have demonstrated that sustaining weight loss after an intervention is often challenging,^16–19^ with OA potentially adding an additional layer of difficulty.^20,21^ The success rate of weight loss sustainability varies widely depending on the study and the population being studied. In a meta-analysis encompassing 29 long-term weight loss studies, it was observed that the majority of participants regained their initial weight before the reduction program within two years, and by the five-year point, over 80% of the lost weight had been regained.^16^ Certain factors that may contribute to weight regain include a pattern of repeated weight loss and gain, difficulties with regulating eating habits, episodes of binge eating, increased feelings of hunger, using food as a coping mechanism for emotional distress, and a tendency to take a less proactive approach when faced with problems.^22^ In addition, factors such as the severity of osteoarthritis, age, comorbidities, and overall health status can impact an individual’s ability to maintain weight loss.^23^

Rates of weight regain are notably elevated within the first year,^17^ with the majority of individuals recovering the lost weight within five years.^14,24^ Quality data revealed that individuals who experienced greater initial weight loss tended to regain weight more quickly. Additionally, while the ongoing availability of weight loss programs to participants beyond the study period was linked to slower weight regain, the offering of financial incentives was associated with a more rapid return of lost weight.^19^

Advancements in anti-obesity pharmacotherapy have introduced drugs such as semaglutide, liraglutide, and tirzepatide, which have shown promising results not only in achieving significant weight loss but also in improving OA-related outcomes.^25–26^ A pivotal randomised controlled trial demonstrated that semaglutide led to an average weight reduction of 13.7% over 68 weeks, significantly greater than the 3.2% observed with placebo. This weight loss was accompanied by substantial improvements in knee OA pain and physical function, suggesting that these drugs could offer dual benefits for individuals with obesity and OA.^25^ A secondary analysis of another weight loss trial found that patients with knee OA treated with liraglutide had greater reduction in weight and improvement in knee-related function compared to placebo.^26^ Furthermore, liraglu-tide after diet-induced weight loss demonstrated a significant weight reduction over 52 weeks, though it did not yield a corresponding improvement in knee pain outcomes.^27^ Additionally, a retrospective cohort study found that the use of anti-obesity medications (AOMs) such as tirzepatide was associated with a 27% lower risk of developing OA compared to non-users.^28^ These findings indicate that AOMs may disrupt the traditional trajectory of weight regain, a persistent challenge in weight management, by not only achieving meaningful weight loss but also potentially altering the underlying biological and mechanical factors that contribute to OA progression.

Overall, while weight loss interventions can be effective for a minority of osteoarthritis patients, it is important to recognise that maintaining weight loss is a long-term process that could not be achieved in every patient with OA and overweight/obesity. While the classical belief is that a combination of diet changes, physical activity, and behaviour modification strategies is often necessary for sustained weight loss and improved health, due to the biological mechanisms that defend the weight set point.^29^ A more effective approach might be to combine efforts to prevent gradual weight gain throughout adulthood, which could help reduce early and irreversible joint damage, as also implicated in the latest recommendations for nonpharmacological management of knee and/or hip osteoarthritis.^4^

Furthermore, a recent Cochrane systematic review and meta-analysis found low to moderate evidence that weight loss interventions provide small to moderate pain relief, slight improvements in physical function, and minimal effects on quality of life. No direct link was observed between the amount of weight lost and better pain or function outcomes.^30^ Therefore, weight loss and weight maintenance alone should not be considered the only indicators of success in managing osteoarthritis in the context of obesity. Instead, healthcare providers should help and motivate patients to adopt healthier dietary habits and increase their physical activity levels if their current habits fall short of national standards.

## RISKS OF WEIGHT LOSS FOR PHYSICAL HEALTH

Defining the optimal pace of weight loss in individuals with knee and hip osteoarthritis can be a challenging task for patients and healthcare providers. There is a lack of consensus among experts about the optimal rate of weight reduction for individuals with ОА, as well as the factors that should be considered when determining the pace.^31^ Some experts advocate for slow and gradual weight loss,^32^ while others recommend more rapid weight loss to achieve rapid pain relief.^24^

Nevertheless, rapid weight loss can lead to joint instability, muscle weakness, and, paradoxically, increased stress on the affected joints in individuals with knee and hip osteoarthritis. Although safety concerns of weight loss are rarely reported, the findings are limited by variations in weight loss methods and inconsistent weight reduction across studies.^30^ Furthermore, a population-based study found that weight loss at a gradual to moderate pace, rather than rapid weight reduction through anti-obesity medications, was associated with decreased all-cause mortality risk in individuals with knee or hip osteoarthritis who are overweight or obese.^32^

### Risk of malnutrition

Knee pain is linked to poor diet quality and malnutrition.^33^ Reducing calorie intake in older adults can further lead to suboptimal nutrient intake or even malnutrition, posing a significant risk.^34^ As calorie needs decrease with age, the requirement for certain nutrients increases, making nutrient-dense diets crucial.^35^ To slow or prevent muscle protein catabolism, protein intake should be maintained or even increased in older individuals restricting calories.^36^ However, the potential for underlying impaired function in high-protein diets can be overlooked, adding to the complexity of nutritional management. Additionally, other dietary approaches, such as low-fat or high-carbohydrate regimens, have been linked to increased knee pain in patients with knee osteoarthritis.^33^ Therefore, promoting a universal weight loss plan without considering these factors may not be the most effective approach.

This highlights the importance of ensuring a high-quality diet through dietary education or counselling to help alleviate knee pain, along with weight management to address imbalances between caloric intake and expenditure. Future prospective studies should explore the direct impact of diet quality on the development of knee pain in individuals, both with and without obesity, and investigate the mechanisms that connect diet quality to knee pain.

### Sarcopenia

Weight loss also results in decreased muscle mass, which can contribute to muscle weakness and decreased stability of the affected joints. A 12-week weight reduction program resulted in a 10.5% weight reduction, a 6.1% loss of lower extremity muscle mass, and a significant decline in muscle strength. Although body weight-normalised muscle strength remained stable, with slight increases of 1.2% and 1.4%, these findings could be of clinical significance.^37^ Strong evidence however exists that individuals with lower levels of quadriceps muscle mass are at an increased risk of developing knee osteoarthritis.^38^ This is because the quadriceps muscle helps absorb shock and distribute weight across the knee joint, reducing the risk of joint damage and degradation. In addition, research has also shown that muscle weakness, including quadriceps weakness, can be a risk factor for knee osteoarthritis, as it can lead to alterations in joint alignment and mechanics that increase the risk of joint damage.^39^ Maintaining adequate quadriceps muscle mass and strength through regular exercise and physical activity can help reduce the risk of knee osteoarthritis and improve joint health. It is always best to discuss any exercise plans with a healthcare provider before starting, especially if you have a history of joint problems or osteoarthritis.

Thus, it is important for individuals with knee and hip osteoarthritis to adopt a gradual weight-loss plan in combination with physical activity and holistic management under the guidance of a healthcare provider. This can help to reduce the risk of joint instability, muscle weakness, and increased stress on the affected joints while still achieving the benefits of weight loss for knee and hip osteoarthritis.

### Joint Instability

Losing weight rapidly can result in a sudden shift in body composition, which can lead to joint instability and increase the risk of falls and injuries.^40^ This can be particularly problematic for individuals with knee and hip osteoarthritis, who may already experience pain, stiffness, and decreased mobility.

It is important to note that the impact of weight loss on joint stability in individuals with osteoarthritis can vary greatly depending on individual factors such as age, overall health status, and the severity of osteoarthritis. In some cases, a slow and gradual approach to weight loss, incorporating both diet and physical activity changes, may be the most effective for reducing the risk of joint instability and improving joint health.^41^

### Osteoporosis and osteopenia

Significant weight loss in older adults can potentially exacerbate bone loss, increasing the risk of osteopenia and osteoporosis, particularly in weight-bearing regions such as the hip.^42^ In fact, osteoporosis and os- teoarthritis are age related disorders with overlapping populations at risk. A few studies that do not focus on osteoarthritis found out that intentional and unintentional weight loss could increase bone loss and hip fracture risk in the population of older women.^43–45^

In a study that measured total hip and femoral neck bone mineral density (BMD) in patients with knee OA at baseline and after 18 months of follow-up, it was found a dose-response relationship between weight loss and reductions in BMD; however, the mean BMD values at the 18-month follow-up remained above the osteopenic threshold for all weight loss categories.^46^ Hypothetically, despite the modest reduction in bone density observed with significant weight loss, the overall positive impact on function and quality of life supports the use of weight loss interventions for managing osteoarthritis,^46^ but is that valid for all patients?

Physical training may help mitigate BMD loss in patients undergoing weight reduction programs. Several studies, including randomised controlled trials (RCT), have explored the combined effects of exercise and caloric restriction on bone health.^47–51^ One study compared exercise plus a very low-calorie diet with other interventions, showing significant reductions in total body BMD only in the group with the greatest weight loss.^49^ Two further RCTs confirmed that combining exercise with caloric restriction lessened hip bone loss and mitigated changes in bone turnover markers, though BMD reductions were still observed.^47,50^ Therefore, optimising weight loss strategies by incorporating appropriate physical training is critical to minimise the negative impact on bone health.

## PSYCHOLOGICAL IMPACT OF WEIGHT LOSS FAILURE AND BODY MASS FLUCTUATIONS

The failure to lose weight to manage knee or hip osteoarthritis can have a significant psychological impact on individuals. Many individuals with knee or hip osteoarthritis may already be struggling with anxiety and depression,^52,53^ and the added stress and frustration of trying to lose weight resulting in failure and weight fluctuations can exacerbate their psychological distress.

In discussing the psychoemotional stress associated with weight loss (over)promotion, it is important to recognise the concept of “slow violence”. This term refers to the gradual, often unnoticed accumulation of harm caused by repeated exposure to weight-loss content, which can have significant emotional and physical impacts.^54^ Such exposure can amplify feelings of stress, frustration, and inadequacy, especially in vulnerable individuals struggling with obesity or osteoarthritis. These subtle but persistent pressures may exacerbate mental health concerns like anxiety or depression. Therefore, it is crucial to develop strategies, including design and policy interventions, that work with affected communities to mitigate the harmful effects of weight-loss messaging and targeted ads. By doing so, health-care providers can offer more compassionate and supportive approaches to weight management.

The negative body image and stigma associated with obesity can lead to feelings of shame, guilt, and low self-esteem, which can further contribute to depression and anxiety.^55^ Additionally, the frustration and disappointment associated with unsuccessful weight-loss attempts can have a negative impact on mental well-being.

While weight loss medications offer promising avenues for addressing obesity and OA,^25–28^ it is essential to consider their broader implications on patient psychology and holistic treatment strategies. For instance, the potential for treatment-related adverse effects, discontinuation rates, and the psychological impact of pharmacological weight-loss efforts must be carefully evaluated. Incorporating these considerations into clinical management plans can help mitigate stress and frustration associated with weight-loss regimens, ultimately fostering a more patient-centred approach.

The pressure to lose weight can also increase stress levels and exacerbate pain and discomfort. The stress of trying to lose weight, combined with the physical demands of exercise and dieting, can further compromise the ability of individuals with knee or hip osteoarthritis to perform daily activities.

**Figure 1.**
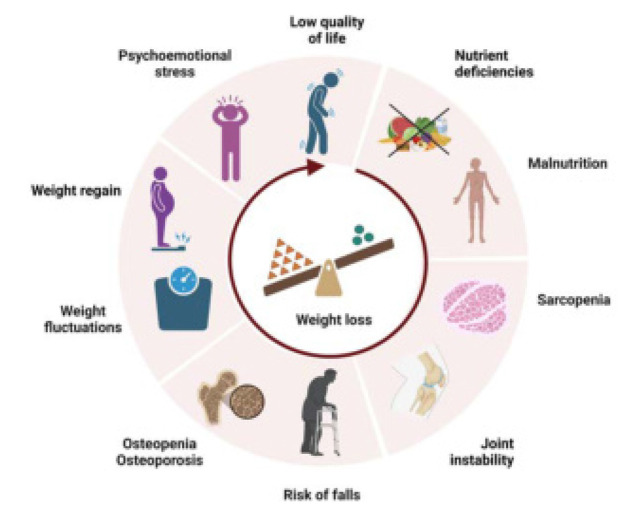
Risks and challenges of weight loss to physical and mental health in patients with osteoarthritis.

It is important for healthcare providers to recognise and address the psychological impact of weight loss advice on individuals with knee or hip osteoarthritis. This may involve referrals to mental health professionals, support groups, and lifestyle modification programs. Providing resources and support to help individuals manage their physical and mental health can help mitigate the negative psychological consequences of weight loss advice and improve their overall quality of life. **Figure 1** highlights the risks and unintended consequences of weight loss in patients with osteoarthritis. These include psychoemotional stress, reduced quality of life, protein, vitamin, and mineral deficiencies, malnutrition, sarcopenia, joint instability, increased risk of falls, osteopenia and osteoporosis, weight fluctuations, and weight regain. The infographic emphasises the importance of balanced and supervised weight management to avoid exacerbating these risks.

## IMPLICATIONS FOR CLINICAL PRACTICE

**Figure 2.**
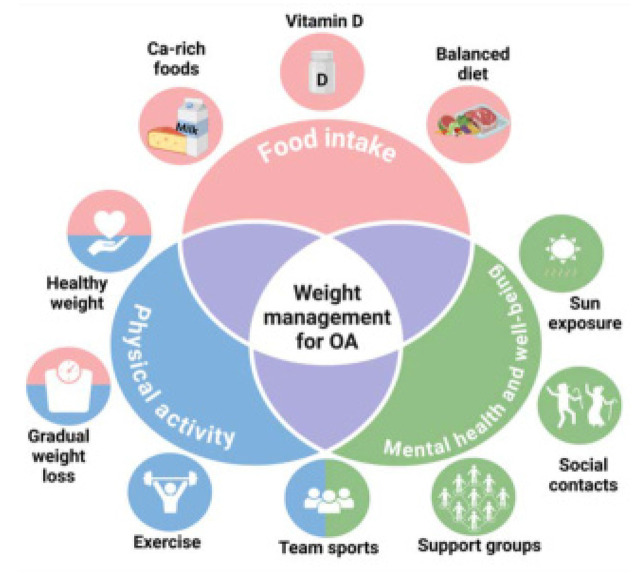
Illustration of a holistic approach and key pillars for balanced weight management in patients with osteoarthritis.

A comprehensive approach to clinical practice is crucial for preventing sarcopenia, osteopenia, osteoporosis, and related complications in elderly individuals with osteoarthritis. Encouraging a diet rich in high-quality proteins supports muscle maintenance and repair, while adequate calcium and vitamin D intake strengthens bone health and reduces fracture risk. Regular physical exercise, including resistance and balance training, improves muscle mass, and joint stability, and reduces the risk of falls. Addressing mental health is equally important, as conditions like depression can exacerbate malnutrition and inactivity. Tailored nutritional guidance to prevent weight fluctuations and mitigate malnutrition ensures that energy needs are met without overburdening compromised joints, promoting overall physical and functional well-being in this population (**Figure 2**). The diagram outlines the three interconnected domains essential for safe and effective weight management in patients with knee or hip osteoarthritis: food intake, physical activity, and mental health and well-being. Each domain includes specific recommendations: ensuring adequate protein, calcium-rich foods, and vitamin D intake, engaging in regular exercise and gradual weight loss strategies, and fostering social connections, team sports, and sun exposure for mental well-being. A multidisciplinary approach is vital to achieve sustainable results without compromising health.

The advent of new anti-obesity therapies represents a paradigm shift in the management of patients with obesity and OA. These therapies not only facilitate significant and sustained weight loss but also potentially address underlying biomechanical and inflammatory pathways^56^ contributing to OA progression. Both glucagon-like peptide (GLP)-1 and dual glucose-dependent insulinotropic polypeptide and GLP-1 receptor agonists work by enhancing insulin secretion, delaying gastric emptying, and promoting satiety, which collectively lead to reduced caloric intake and weight loss. A recent meta-analysis demonstrated that these agents can reduce body weight by over 10%,^57^ with associated improvements in physical function and quality of life in patients with knee OA.^25,26^ Furthermore, evidence suggests these drugs may lower the risk of OA development in individuals with obesity,^28^ likely by mitigating joint overload and systemic inflammation. The long-term use of these therapies could transform the clinical approach to OA by targeting obesity as a modifiable risk factor. By integrating these medications into comprehensive treatment plans, clinicians may not only improve weight-related outcomes but also reduce OA symptoms, delay disease progression, and enhance patient adherence to long-term management strategies. While these drugs could represent a game-changing addition to the management of obesity and OA, their long-term impact on weight maintenance and OA outcomes warrants further investigation.

Based on our comprehensive review of data from designated databases, we identified key concerns, side effects, and challenges associated with failure to lose weight, as well as the related risks. These findings, together with clinical implications, are synthesised and presented in **Table 1**.

**Table 1.** Key concerns, side effects, and risks of weight loss in patients with osteoarthritis.

**Key Concern**	**Side Effects & Risks**	**Clinical Implications**
**Weight Regain**	High rates of weight regain post-intervention, often within 1-5 years, with up to 80% of lost weight regained. Factors include emotional eating, binge eating, and poor diet adherence.	Weight management requires long-term follow- up, personalised behavioural therapy, and strategies for sustainable lifestyle changes.
**Malnutrition**	Caloric restriction, especially in older adults, can lead to nutrient deficiencies or malnutrition, potentially exacerbating osteoarthritis symptoms.	Regular nutritional assessments are necessary, with an emphasis on ensuring sufficient intake of essential nutrients such as protein, vitamins, and minerals.
**Sarcopenia**	Weight loss can lead to muscle mass reduction, increasing the risk of muscle weakness, joint instability, and progression of osteoarthritis.	Incorporating resistance training into weight loss plans can help preserve muscle mass and strength, crucial for joint stability and overall mobility.
**Joint Instability**	Rapid weight loss can cause changes in body composition that may lead to instability in joints, increased risk of falls, and injuries.	Slow, controlled weight loss combined with exercises to improve balance and strength can mitigate the risk of falls and joint instability.
**Osteoporosis & Osteopenia**	Weight loss, especially in older adults, can accelerate bone loss, increasing the risk of osteopenia or osteoporosis, particularly in weight-bearing regions such as the hips.	Patients should be monitored for bone density loss. Weight loss interventions should include strategies to minimise bone loss, such as calcium and vitamin D supplementation.
**Psychological Impact**	Failure to achieve sustained weight loss or recurrent weight fluctuations can lead to psychological distress, including anxiety and depression.	Provide psychological support and counseling to address the mental health challenges associated with weight management and osteoarthritis.

## CONCLUSIONS

While weight reduction was found beneficial for most patients with knee and hip OA, potential concerns and detriments should be considered, while identifying certain risk groups for weight loss programs. Substantial weight reduction might not be realistic for each patient with OA, while weight sustainability after successful intervention is not guaranteed because of biological weight set point. Therefore, of most importance is the maintenance of healthy body mass to preserve joint health and structure. Drastic weight reduction may lead to an increased risk of sarcopenia, osteoporosis, and falls in already compromised stability due to osteoarthritis; therefore, physical activity should be of utmost importance and recommendation.

Failure to lose weight and frequent fluctuations for individuals with knee or hip osteoarthritis might be detrimental to patients’ psychoemotional state and well-being and should be further investigated. Pharmacological anti-obesity therapies show promise in achieving significant weight loss and improving certain outcomes in OA, though their impact on pain and long-term weight maintenance requires further investigation.

Healthcare providers can enhance the effectiveness of weight loss advice and improve overall quality of life by offering support, identifying patient groups that may benefit most, and providing resources to help individuals manage their weight at optimal level. This holistic approach helps to minimise the potential risks associated with weight loss programs and recommendations.

## AUTHOR CONTRIBUTIONS

TG and PK contributed to the study’s conception, data acquisition, and analysis, as well as the interpretation of findings. They critically revised the manuscript, approved the final version for publication, and agreed to be accountable for all aspects of the work, ensuring its accuracy and integrity. Both authors take full responsibility for the integrity and accuracy of the work.

## FUNDING

No specific funding was received regarding this work.

## CONFLICT OF INTEREST

The authors declare no conflicts of interest related to this work.

## DISCLAIMER

No part of this manuscript is copied or published elsewhere in whole or in part.

## References

[1] Martel-Pelletier J Barr AJ Cicuttini FM Conaghan PG Cooper C Goldring MB Osteoarthritis. Nat Rev Dis Primers 2016;2:16072. 27734845 10.1038/nrdp.2016.72

[2] Bierma-Zeinstra SMA Koes BW. Risk factors and prognostic factors of hip and knee osteoarthritis. Nat Clin Pract Rheumatol 2007;3(2):78–85. 17299445 10.1038/ncprheum0423

[3] Georgiev T Angelov AK. Modifiable risk factors in knee osteoarthritis: treatment implications. Rheumatol Int 2019;39(7):1145–57. 30911813 10.1007/s00296-019-04290-z

[4] Moseng T Vliet Vlieland TPM Battista S Beckwée D Boyadzhieva V Conaghan PG EULAR recommendations for the non-pharmacological core management of hip and knee osteoarthritis: 2023 update. Ann Rheum Dis 2024;83(6):730–40. 38212040 10.1136/ard-2023-225041 PMC11103326

[5] Kolasinski SL Neogi T Hochberg MC Oatis C Guyatt G Block J 2019 American College of Rheumatology/Arthritis Foundation Guideline for the Management of Osteoarthritis of the Hand, Hip, and Knee. Arthritis Care Res (Hoboken) 2020;72(2):149–62. 31908149 10.1002/acr.24131 PMC11488261

[6] Mazzei DR Ademola A Abbott JH Sajobi T Hildebrand K Marshall DA. Are education, exercise and diet interventions a cost-effective treatment to manage hip and knee osteoarthritis? A systematic review. Osteoarthritis Cartilage 2021;29(4):456–70. 33197558 10.1016/j.joca.2020.10.002

[7] Panunzi S Maltese S De Gaetano A Capristo E Bornstein SR Mingrone G. Comparative efficacy of different weight loss treatments on knee osteoarthritis: A network meta-analysis. Obes Rev 2021;22(8):e13230. 33855769 10.1111/obr.13230

[8] Robson EK Hodder RK Kamper SJ O’Brien KM Williams A Lee H Effectiveness of Weight-Loss Interventions for Reducing Pain and Disability in People With Common Musculoskeletal Disorders: A Systematic Review With Meta-Analysis. J Orthop Sports Phys Ther 2020;50(6):319–33. 32272032 10.2519/jospt.2020.9041

[9] Gersing AS Solka M Joseph GB Schwaiger BJ Heilmeier U Feuerriegel G Progression of cartilage degeneration and clinical symptoms in obese and overweight individuals is dependent on the amount of weight loss: 48-month data from the Osteoarthritis Initiative. Osteoarthritis Cartilage 2016;24(7):1126–34. 26828356 10.1016/j.joca.2016.01.984 PMC4907808

[10] Jin X Gibson AA Gale J Schneuer F Ding D March L Does weight loss reduce the incidence of total knee and hip replacement for osteoarthritis? A prospective cohort study among middle-aged and older adults with overweight or obesity. Int J Obes (Lond) 2021;45(8):1696–704. 33993192 10.1038/s41366-021-00832-3 PMC8310800

[11] Giardullo L Corrado A Maruotti N Cici D Mansueto N Cantatore FP. Adipokine role in physiopathology of inflammatory and degenerative musculoskeletal diseases. Int J Immunopathol Pharmacol 2021;35:20587384211015034. 33983056 10.1177/20587384211015034 PMC8127732

[12] Gasparyan AY Ayvazyan L Blackmore H Kitas GD. Writing a narrative biomedical review: considerations for authors, peer reviewers, and editors. Rheumatol Int 2011;31(11):1409–17. 21800117 10.1007/s00296-011-1999-3

[13] Thivel D Ennequin G Lambert C Siroux J Ratel S Boscaro A Improved walking energy efficiency might persist in presence of simulated full weight regain after multidisciplinary weight loss in adolescents with obesity: the POWELL study. Int J Obes (Lond) 2024;48(3):384–93. 38052874 10.1038/s41366-023-01427-w

[14] Machado AM Guimarães NS Bocardi VB da Silva TPR Carmo ASD Menezes MC Understanding weight regain after a nutritional weight loss intervention: Systematic review and meta-analysis. Clin Nutr ESPEN 2022;49:138–53. 35623805 10.1016/j.clnesp.2022.03.020

[15] Hinte LC Castellano-Castillo D Ghosh A Melrose K Gasser E Noé F Adipose tissue retains an epigenetic memory of obesity after weight loss. Nature 2024;636:457–65. 39558077 10.1038/s41586-024-08165-7 PMC11634781

[16] Anderson JW Konz EC Frederich RC Wood CL. Long-term weight-loss maintenance: a meta-analysis of US studies. Am J Clin Nutr 2001;74(5):579–84. 11684524 10.1093/ajcn/74.5.579

[17] Weiss EC Galuska DA Kettel Khan L Gillespie C Serdula MK. Weight regain in U.S. adults who experienced substantial weight loss, 1999–2002. Am J Prev Med 2007;33(1):34–40. 17572309 10.1016/j.amepre.2007.02.040

[18] Wadden TA Butryn ML Byrne KJ. Efficacy of lifestyle modification for long-term weight control. Obes Res 2004;12 Suppl:151S–62S. 15687411 10.1038/oby.2004.282

[19] Hartmann-Boyce J Theodoulou A Oke JL Butler AR Scarborough P Bastounis A Association between characteristics of behavioural weight loss programmes and weight change after programme end: systematic review and meta-analysis. BMJ 2021;374:n1840. 34404631 10.1136/bmj.n1840 PMC8369384

[20] Smith TO Purdy R Lister S Salter C Fleetcroft R Conaghan PG. Attitudes of people with osteoarthritis towards their conservative management: a systematic review and meta-ethnography. Rheumatol Int 2014;34(3):299–313. 24306266 10.1007/s00296-013-2905-y

[21] Sariyildiz A Coskun Benlidayi I Olmez Engizek S Deniz V. The relation of psychological status and type D personality with central sensitization in knee osteoarthritis: everything is in your mind! Rheumatol Int 2023;43(12):2261–9. 37776500 10.1007/s00296-023-05471-7

[22] Elfhag K Rössner S. Who succeeds in maintaining weight loss? A conceptual review of factors associated with weight loss maintenance and weight regain. Obes Rev 2005;6(1):67–85. 15655039 10.1111/j.1467-789X.2005.00170.x

[23] Georgiev T Dimitrov S Kabakchieva P. AGING GRACEFULLY IN OSTEOARTHRITIS: IMPACT OF COMORBIDITIES. Anti-Aging Eastern Europe 2024;3(3):124–34.

[24] Flore G Preti A Carta MG Deledda A Fosci M Nardi AE Weight Maintenance after Dietary Weight Loss: Systematic Review and Meta-Analysis on the Effectiveness of Behavioural Intensive Intervention. Nutrients 2022;14(6). 10.3390/nu14061259 PMC8953094 35334917

[25] Bliddal H Bays H Czernichow S Uddén Hemmingsson J Hjelmesæth J Hoffmann Morville T Once-Weekly Semaglutide in Persons with Obesity and Knee Osteoarthritis. N Engl J Med 2024;391(17):1573–83. 39476339 10.1056/NEJMoa2403664

[26] Bartholdy C Overgaard A Gudbergsen H Bliddal H Kristensen LE Henriksen M. Changes in physical activity during a one-year weight loss trial with liraglutide vs placebo in participants with knee osteoarthritis: Secondary analyses of a randomised controlled trial. Osteoarthr Cartil Open 2022;4(2):100255. 36475294 10.1016/j.ocarto.2022.100255 PMC9718081

[27] Gudbergsen H Overgaard A Henriksen M Wæhrens EE Bliddal H Christensen R Liraglutide after diet-induced weight loss for pain and weight control in knee osteoarthritis: a randomized controlled trial. Am J Clin Nutr 2021;113(2):314–23. 33471039 10.1093/ajcn/nqaa328

[28] Baser O Rodchenko K Vivier E Baser I Lu Y Mohamed M. The impact of approved anti-obesity medications on osteoarthritis. Expert Opin Pharmacother 2024;25(11):1565–73. 39129529 10.1080/14656566.2024.2391524

[29] Cicuttini FM Proietto J Lim YZ. Our biology working against us in obesity: A narrative review on implications for management of osteoarthritis. Osteoarthr Cartil Open 2023;5(4):100407. 37744021 10.1016/j.ocarto.2023.100407 PMC10514453

[30] Juhl CB Christensen R Bolvig J Bartels EM Astrup A Singh J Weight loss for overweight patients with knee or hip osteoarthritis: Cochrane systematic review and meta-analysis of randomized trials. Osteoarthritis Cartilage 2024;32:S225.

[31] Lim YZ Wong J Hussain SM Estee MM Zolio L Page MJ Recommendations for weight management in osteoarthritis: A systematic review of clinical practice guidelines. Osteoarthr Cartil Open 2022;4(4):100298. 36474793 10.1016/j.ocarto.2022.100298 PMC9718266

[32] Wei J Hunter D Lane NE Wu J Zeng C Lei G Weight Loss Induced by Antiobesity Medications and All-Cause Mortality Among Patients With Knee or Hip Osteoarthritis. Arthritis Rheumatol 2024;76(4):577–86. 38053480 10.1002/art.42754

[33] Shin WY Kim JH. Poor diet quality is associated with self-reported knee pain in community-dwelling women aged 50 years and older. PLoS One 2021;16(2):e0245630. 33591989 10.1371/journal.pone.0245630 PMC7886155

[34] MillerSLWolfeRR. The danger of weight loss in the elderly. J Nutr Health Aging 2008;12(7):487–91.18615231 10.1007/BF02982710

[35] Troesch B Biesalski HK Bos R Buskens E Calder PC Saris WHM Increased Intake of Foods with High Nutrient Density Can Help to Break the Intergenerational Cycle of Malnutrition and Obesity. Nutrients 2015;7(7):6016–37. 26197337 10.3390/nu7075266 PMC4517043

[36] Phillips SM Paddon-Jones D Layman DK. Optimizing Adult Protein Intake During Catabolic Health Conditions. Adv Nutr 2020;11(4):S1058–69. 32666115 10.1093/advances/nmaa047 PMC7360447

[37] Kim B Tsujimoto T So R Zhao X Oh S Tanaka K. Changes in muscle strength after diet-induced weight reduction in adult men with obesity: a prospective study. Diabetes Metab Syndr Obes 2017;10:187–94. 28533692 10.2147/DMSO.S132707 PMC5431739

[38] Toda Y Segal N Toda T Kato A Toda F. A decline in lower extremity lean body mass per body weight is characteristic of women with early phase osteoarthritis of the knee. J Rheumatol 2000;27(10):2449–54. 11036843

[39] Gong Z Li J He Z Li S Cao P Ruan G Quadriceps strength is negatively associated with knee joint structural abnormalities-data from osteoarthritis initiative. BMC Musculoskelet Disord 2022;23(1):784. 35978313 10.1186/s12891-022-05635-9 PMC9382744

[40] Xu C Ebeling PR Scott D. Body composition and falls risk in older adults. Curr Geriatr Rep 2019;8(3):210–22.

[41] Sherrington C Fairhall N Kwok W Wallbank G Tiedemann A Michaleff ZA Evidence on physical activity and falls prevention for people aged 65+ years: systematic review to inform the WHO guidelines on physical activity and sedentary behaviour. Int J Behav Nutr Phys Act 2020;17(1):144. 33239019 10.1186/s12966-020-01041-3 PMC7689963

[42] Langlois JA Mussolino ME Visser M Looker AC Harris T Madans J. Weight loss from maximum body weight among middle-aged and older white women and the risk of hip fracture: the NHANES I epidemiologic follow-up study. Osteoporos Int 2001;12(9):763–8. 11605743 10.1007/s001980170053

[43] Ensrud KE Ewing SK Stone KL Cauley JA Bowman PJ Cummings SR Intentional and unintentional weight loss increase bone loss and hip fracture risk in older women. J Am Geriatr Soc 2003;51(12):1740–7. 14687352 10.1046/j.1532-5415.2003.51558.x

[44] Compston JE Wyman A FitzGerald G Adachi JD Chapurlat RD Cooper C Increase in Fracture Risk Following Unintentional Weight Loss in Postmenopausal Women: The Global Longitudinal Study of Osteoporosis in Women. J Bone Miner Res 2016;31(7):1466–72. 26861139 10.1002/jbmr.2810 PMC4935593

[45] Soltani S Hunter GR Kazemi A Shab-Bidar S. The effects of weight loss approaches on bone mineral density in adults: a systematic review and meta-analysis of randomized controlled trials. Osteoporos Int 2016;27(9):2655–71. 27154437 10.1007/s00198-016-3617-4

[46] Messier SP Resnik AE Beavers DP Mihalko SL Miller GD Nicklas BJ Intentional Weight Loss in Overweight and Obese Patients With Knee Osteoarthritis: Is More Better? Arthritis Care Res (Hoboken) 2018;70(11):1569–75. 29911741 10.1002/acr.23608 PMC6203601

[47] Armamento-Villareal R Sadler C Napoli N Shah K Chode S Sinacore DR Weight loss in obese older adults increases serum sclerostin and impairs hip geometry but both are prevented by exercise training. J Bone Miner Res 2012;27(5):1215–21. 22392834 10.1002/jbmr.1560 PMC3361603

[48] Villareal DT Chode S Parimi N Sinacore DR Hilton T Armamento-Villareal R Weight loss, exercise, or both and physical function in obese older adults. N Engl J Med 2011;364(13):1218–29. 21449785 10.1056/NEJMoa1008234 PMC3114602

[49] Haywood CJ Prendergast LA Purcell K Le Fevre L Lim WK Galea M Very Low Calorie Diets for Weight Loss in Obese Older Adults-A Randomized Trial. J Gerontol A Biol Sci Med Sci 2017;73(1):59–65. 28329121 10.1093/gerona/glx012

[50] Villareal DT Shah K Banks MR Sinacore DR Klein S. Effect of weight loss and exercise therapy on bone metabolism and mass in obese older adults: a one-year randomized controlled trial. J Clin Endocrinol Metab 2008;93(6):2181–7. 18364384 10.1210/jc.2007-1473 PMC2435639

[51] Shah K Armamento-Villareal R Parimi N Chode S Sinacore DR Hilton TN Exercise training in obese older adults prevents increase in bone turnover and attenuates decrease in hip bone mineral density induced by weight loss despite decline in bone-active hormones. J Bone Miner Res 2011;26(12):2851–9. 21786319 10.1002/jbmr.475 PMC3206995

[52] Stubbs B Aluko Y Myint PK Smith TO. Prevalence of depressive symptoms and anxiety in osteoarthritis: a systematic review and meta-analysis. Age Ageing 2016;45(2):228–35. 26795974 10.1093/ageing/afw001

[53] Hall M Dobson F Van Ginckel A Nelligan RK Collins NJ Smith MD Comparative effectiveness of exercise programs for psychological well-being in knee osteoarthritis: A systematic review and network meta-analysis. Semin Arthritis Rheum 2021;51(5):1023–32. 34416624 10.1016/j.semarthrit.2021.07.007

[54] Gak L Olojo S Salehi N. The distressing ads that persist: Uncovering the harms of targeted weight-loss ads among users with histories of disordered eating. Proc ACM Hum Comput Interact 2022;6(CSCW2):1–23.37360538

[55] Puhl RM Heuer CA. Obesity stigma: important considerations for public health. Am J Public Health 2010;100(6):1019–28. 20075322 10.2105/AJPH.2009.159491 PMC2866597

[56] Holst JJ. GLP-1 physiology in obesity and development of incretin-based drugs for chronic weight management. Nat Metab 2024;6(10):1866–85. 39160334 10.1038/s42255-024-01113-9

[57] Pan XH Tan B Chin YH Lee ECZ Kong G Chong B Efficacy and safety of tirzepatide, GLP-1 receptor agonists, and other weight loss drugs in overweight and obesity: a network meta-analysis. Obesity (Silver Spring) 2024;32(5):840–56. 38413012 10.1002/oby.24002

